# Evaluation of antibacterial activity of enterocin A-colicin E1 fusion peptide

**DOI:** 10.22038/ijbms.2020.47826.11004

**Published:** 2020-11

**Authors:** Hadis Fathizadeh, Mahmood Saffari, Davoud Esmaeili, Rezvan Moniri, Morteza Salimian

**Affiliations:** 1 Department of Microbiology and Immunology, Faculty of Medicine, Kashan University of Medical Sciences, Kashan, Iran; 2 Department of Microbiology and Applied Microbiology Research Center, Systems Biology and Poisonings Institute, Baqiyatallah University of Medical Sciences, Tehran, Iran; 3 Applied Virology Research Center, Baqiyatallah University of Medical Sciences, Tehran, Iran; 4 Anatomical Science Research Center, Kashan University of Medical Sciences, Kashan, Iran

**Keywords:** Antibacterial activity, Bacteriocins, Colicin E1, Enterocin A, Fusion peptide

## Abstract

**Objective(s):**

Bacterial resistance to most common antibiotics is a harbinger of the requirement to find novel anti-infective, antimicrobials agents, and increase innovative strategies to struggle them. Numerous bacteria produce small peptides with antimicrobial activities called bacteriocin. This study aimed to investigate the antibacterial properties of the fusion protein of Enterocin A and Colicin E1 modified against pathogens.

**Materials and Methods:**

Analysis of recombinant bacteriocin Enterocin A and Colicin E1 (ent A-col E1) was performed to assay the stability and antibacterial activity of this fusion protein. The pET-22b vector was employed to express the coding sequence of the ent A-col E1 peptide in *Escherichia coli* BL21 (DE3). Minimum inhibitory concentration (MIC), disk diffusion, and time-kill tests were performed to evaluate the antibacterial activity of the ent A-col E1 against *Pseudomonas aeruginosa* (ATCC 9027), *Escherichia coli* (ATCC 10536), *Enterococcus faecalis* (ATCC 29212), and *Staphylococcus aureus* (ATCC 33591).

**Results:**

The suggested recombinant peptide had good antibacterial activity against both Gram-negative and Gram-positive pathogens. It has also good stability at various temperatures, pH levels, and salt concentrations.

**Conclusion:**

Because bacteriocins are harmless compounds, they can be recommended as therapeutic or preventive supplements to control pathogens. According to the obtained results, the ent A-col E1 peptide can serve as an efficient antibacterial compound to treat or prevent bacterial infections.

## Introduction

Antimicrobial resistance is a dilemma, which has recently raised concerns worldwide. The application of antibiotics in the treatment of infectious diseases resulted in the emergence of multidrug-resistant bacteria (1); to overcome this problem, new antimicrobial agents should be developed (2). The use of a mixture of antimicrobial factors on different infections may also reduce the emergence of resistant strains of bacteria in hospitals, aiming at reducing the administration of therapeutic doses (3). Bacteriocins are good candidates for antibacterial therapy (4, 5). Both lipoteichoic acids (LTA) and lipopolysaccharide (LPS) from Gram-positive and Gram-negative bacteria, respectively, prepare binding sites at outer membrane surfaces that enable cationic antimicrobial peptides to attain the target cytoplasmic membrane. Electrostatic interactions between the cationic molecules and negatively-charged sites on the outer membrane of bacteria facilitate the attachment of antimicrobial peptides to these membranes. Bacteriocins, peptides, or proteins synthesized by ribosomes have a diverse range of antimicrobial activity and are produced by a broad group of bacteria. Given the urgent need of the present world to find effective ways of controlling pathogenic bacteria, the study of bacteriocins as an alternative antibiotic candidate is important (6). 

A large group of bacteria living as microflora in humans and animals’ intestinal tracts is called lactic acid bacteria (LAB) that are potent in producing bacteriocins (7). *Enterococci* are LABs producing a bacteriocin called enterocin; it can be used as an antimicrobial agent in the control of pathogenic microorganisms in clinical settings and food industry. Enterocins are mainly produced by *Enterococcus fascium*, *E. faecalis*, and *E. mondii* and grow out of the medial membrane environment *in vitro*. Bacteriocins produced by *Enterococcus* include bacteriocin 35, enterocin A, B, L50A/B, and P, which belong to class II bacteriocins (4). Enterocin A of *E. fascium* has an isoelectric point of about 10, which is possibly a cyclic peptide. Therefore, given the peptide cyclicity and the effective mechanism of the attack on other pathogens, as well as the remarkable stability of this peptide, it is a good candidate for protecting pathogens and treating cancer (8). Enterocin exerts its antimicrobial activity on both Gram-positive and -negative bacteria, while most LAB-derived bacteriocins affect LAB and other Gram-positive bacteria (9). A variety of investigations have reported a broad range of the antibacterial function of entercins against different bacteria such as *Listeria monocytogenes *and* Staphylococcus aureus* (4, 9-12). Enterocin A is a heat-stable bacteriocin and has antibacterial activity against foodborne pathogenic microorganisms (4). Since *Enterococci* carry resistance genes and potential virulence factors, the production of enterocins by the safe bacterial host has recently drawn much attention (13).

Colicins are Gram-negative derived bacteriocins, which are also used as a model for ecology, function, evolution, structure, and genetic organization of bacteriocin nowadays (14). Colicins are toxic proteins of high molecular mass that are made by colicinogenic strains of *Escherichia coli* and other relevant species of Enterobacteriaceae. The widespread use of antibiotics today has created resistant strains and is one of the challenges of the new century. Concerns about antibiotic resistance are so serious that in many countries huge funds have been devoted to the discovery of pharmaceutical alternatives (15). Col E1 bacteriocin forms ion channels and depolarizes cytoplasmic membrane and, therefore, is cytotoxic to *E. coli* and the closely related species (16). 

Genetic engineering has been used to enhance the function of bacteriocins in various studies. One instance of these new constructs includes the merger of a channel forming colicin Ia and a pheromone (agrD) constructed by *S. aureus*. *In vitro* studies showed that the growth of wild type and two antibiotic-resistant strains of *S. aureus* is limited by this fusion product. (17, 18). Also, another study showed that the bioengineered nisin A derivatives demonstrated better antimicrobial function than nisin when tested against several Gram-negative food-related pathogens (19). Combining two antimicrobial agents with different mechanisms against pathogenic bacteria is an effective approach to prevent the progression of infectious diseases caused by antibiotic-resistant bacteria (3). Results of a study by Ankaiah *et al.* showed that the heterodimer of enterocin-A+B exerts antibacterial and anti-biofilm activities against *S. aureus*, *L. monocytogenes, E. coli,* and *Salmonela enterica *(20). According to studies, recombinant peptides have higher expression in new hosts, and intensified antibacterial properties can be obtained and evaluated using bioinformatics designs. 

 We designed a recombinant peptide (ent A-col E1) consisting of enterocin A and colicin E1 to enhance antibacterial potency and reported the bioinformatics design and analysis, expression optimization, purification, bioactivity, and subsequent assessment of the antibacterial activity of ent A-col E1 against Gram-positive and Gram-negative bacterial cells. Furthermore, we evaluated the activity of chimeric peptide in various conditions.

## Materials and Methods

***Design and analysis of the recombinant peptide***

The sequences of enterocin A (Gene ID: 13001078, www.ncbi.nlm.nih.gov/gene/13001078) and colicin E1 (Gene ID: 2693957, www.ncbi.nlm.nih.gov/gene/2693957) were obtained from the GenBank database. A linker sequence including cysteine was used between the two bacteriocins to maintain the structure and flexibility of the final structure. The designed sequence was assessed by the Basic Local Alignment Search Tool (BLAST) (http://blast.ncbi.nlm.nih.gov/Blast.cgi). 

***Physiochemical characterization***

For physicochemical characterization, molecular weight, theoretical IP (isoelectric point), EI (extinction coefficient), AI (aliphatic index), II (instability index), and GRAVY (grand average hydropathy) were estimated by the Expasy’s ProtParam server for the recombinant bacteriocin (ent A-col E1) (http://us.expasy.org/tools/ protparam.html). The hydrophobicity plot of the ent A-colE1 continuous amino acid residues was specified by creating the Kyte-Doolittle hydropathy graph (https://web.expasy.org/cgi-bin/protscale/protscale.pl).

***Secondary and tertiary structure prediction***

Secondary structures prediction of the ent A-col E1 was performed by the GOR4 secondary structure prediction method (https://npsa-prabi.ibcp.fr/cgibin/npsa) that indicates the sequences arrangements as α-helices, β-sheets, and coils structures. Then, the I-TASSER server was employed to model the tertiary structure in PDB format. Using multiple threading alignments, Quality I-TASSER produces 3D models as well as their confidence score (i.e. C-score). For model stability studies, 3D PDB models were evaluated by Ramachandran plot analysis (RAMPAGE software). ProSA-web was also used to qualitatively assess the structures of the ent A-col E1 peptide deposited in PDB and the results were plotted based on Z-score.

***Molecular dynamic simulation***

Molecular dynamic (MD) simulation was processed in explicit aqueous solution for 100 nsec. The complex was located in a cubic box with an edge of 1.2 nm. Next, the box dissolved with molecules of simple-point-charge (SPC) water . To neutralize the system completely, 5 ions were included. Minimization of energy was applied to the complex species. It followed with 100 psec Isothermal–isobaric (NPT) and 100 psec NVT. The MD simulation at 310 k was performed with a position on protein. Eventually, simulation of MD was performed on the total system for 100 nsec. Van der Waals force cut-off distance was 1.2 nm. Pressure and temperature coupling was performed by the Parrinello-Rahman method. 310 K was considered as a reference temperature. Every 10 psec, the trajectory was recorded with a time step of 2 fsec. The simulation was performed by GROMACS 5.1.4 package (ref). The GROMOS96 53a1 force field validated and effectively applied in various systems such as proteins. The root-mean-square deviation (RMSD) analysis was performed to determine the stability of the ent A-col E1 peptide for 100 nsec. RMSD analysis shows the difference between the value predicted by the model and the actual value. 

***Synthesis of the coding gene fragment and transformation***

The synthetic construct (BIOMATIK, Canada) was composed of the coding sequence of the enterocin A, colicin E1, and linker inserted between Bam H1 and Sac1 restriction sites of the pET-22b plasmid encoding His-tag. The *E. coli* strain BL21 was grown in Luria-Bertani (LB) medium at 37 ^º^C. The LB medium was then augmented with ampicillin (100 μg/ml) for plasmid selection. Ent A-col E1 construct was transformed into *E. coli* BL21 cells. The transformation process was confirmed using colony PCR of randomly picked colonies (21). 

***Expression, purification, and confirmation of ent A-col E1 peptide***

When the expression of the ent A-col E1 gene was induced with IPTG (isopropyl b-D-1-thiogalactopyranoside), its overexpression was optimized using IPTG at different concentrations and incubation times as 2, 3, 4, 5, and 24 hr. Then, 15% sodium dodecyl sulfate-polyacrylamide gel electrophoresis (SDS-PAGE) was used to assess the expression of ent A-col E1, which was then confirmed by Western blotting using Anti-His tag-specific monoclonal antibody (22). Then, ent A-col E1 expression cultures of BL21 were centrifuged and the pellets were lysed by sonication. In the next stage, the expressed peptide was purified by Ni-NTA column with a denaturation system based on His tag affinity chromatography and by using Triton X-114 for removing the LPS of the fusion proteins (23). LPS level of the purified peptide was measured using the chromogenic Limulus Amebocyte Lysate test (LAL), according to the manufacturer’s protocol (Lonza). Then, the purified peptide was dialyzed against different concentrations of urea. Bradford assay was utilized to measure the final concentration with a concentrated Bradford solution (24).

***Disk diffusion assay***

Antibacterial activity of recombinant peptide was tested *in vitro* on four bacterial strains. All isolates were purchased from Iran National Genetic and Biological Resources Center. Because the recombinant peptide is not present in the CLSI, the results were compared with similar concentrations of an antibiotic. 

 Sterile blank disks were soaked in 5, 10, 30, and 110 µg of the ent A-col E1 peptide in PBS and the negative control ones in PBS. In the case of each strain, only one concentration proportional to the concentration of a common antibiotic used for that strain was used. For *S. aureus*, *penicillin* disc (10 units), and 10 µg of ent A-col E1 peptide were used to examine the diameter of the inhibitory halo. Disks containing *Piperacillin/tazobactam* (110 μg) and 110 μg of ent A-col E1 peptide were used for *Pseudomonas aeruginosa*. Disks containing *cefazolin* (30 μg) and 30 μg of ent A-col E1 peptide were applied for *E. coli*. Disks containing *ampicillin* )10 μg) and 10 μg of ent A-col E1 peptide were used for *E. faecalis*. After drying the discs at room temperature, the disk diffusion assay was performed by Kirby-Bauer method on Muller Hinton agar for *P. aeruginosa, E. coli, E. faecalis, *and *S. aureus*. The prepared disks were spotted onto Mueller-Hinton agar (25). The plates were incubated for 20 hr at 37 ^°^C. Then, the inhibition zone diameter around the recombinant peptide disks was measured. The procedure was repeated in triplicate for each strain and their mean inhibition zones were calculated.

***Minimum inhibitory concentration assays***

Minimum inhibitory concentration (MIC) of the ent A-col E1 peptide was determined by a microdilution method. A total of 100 ml cation-controlled Muller Hinton broth was divided into each well and twofold dilutions of the recombinant peptide were made (CLSI, 2016) (26, 27). Different concentrations (0, 5, 10, 20, 40, 80, 160 μg/ml) of the ent A-col E1 peptide and *P. aeruginosa, E. coli, S. aureus, *and *E. faecalis *were used in this experiment. Each microtitre well was inoculated with 10 μl of the bacterial inoculum of 5×10^5 ^CFU ml^-1^ and the plates were incubated at 37 ^º^C for 18 hr under aerobic conditions. The lowest concentration of a compound that can completely inhibit bacterial growth (>90%) as detected at OD_600_, compared to the control group, is considered as MIC. This assay was repeated in triplicate and their average was calculated.

***Time–kill curve analysis***

To investigate the rate and extent of bacterial reduction when treated with a compound, a time-kill curve (CFU as a function of time) was created. The experiments were conducted in Muller Hinton broth for 12 hr with the 0.5×MIC, 1×MIC, and 2×MIC of the ent A-col E1 peptide used against all the four strains. An initial inoculum of the approximately mid-log phase of bacterial growth (OD_590_ nm∼0.5–0.6) was taken for all the experiments. Samples (0.1 ml) were collected on 0, 2, 4, 6, 8, and 12 hr and aliquoted in duplicate onto Muller–Hinton agar plates. These plates were then incubated at 37 ^º^C for 24 hr (28). The colony count was performed (10–100 per plate), representing a lower detection limit of 10^2^ CFU ml^-1^. 

***Effects of NaCl concentration, heat, and pH treatments on the ent A-col E1 peptide activity***

MIC values of ent A-col E1 peptide (10 μg/ml) ​​were used to evaluate its stability at different physiological conditions. The tolerability of ent A-col E1 peptide in different concentrations of NaCl, temperature, and pH was measured in mid-log phase cultures (OD_590_ nm ∼0.6) of *S. aureus* as a model strains. Sodium acetate buffer (50 mmol/l; pH 7.5) was prepared to contain various concentrations of NaCl from 0 to 150 mmol/l. Microplates containing bacterial culture, recombinant peptide, and 100 µl of different salt concentrations (0, 50, 100, or 150 mM NaCl) were incubated for 30 min. The turbidity of the wells was then read by spectrophotometer in OD_590_ nm (21). 

To assay the thermal stability of ent A-col E1 peptide, it was treated under a span of temperatures from 5 to 60 ^º^C for 15 min. Then, ent A-col E1 peptide was added into *S. aureus* culture and the maintain of bacteriocin activity was determined by reading the turbidity of the wells using spectrophotometer in OD_590_ nm. In the next step, ent A-col E1 peptide was treated with pH values ranging from 4 to 12 by adding appropriate volumes of 4 N HCl or 4 N NaOH for 15 min. Next, the antibacterial activity was determined by reading the turbidity of the wells using spectrophotometer in OD_590_ nm (21). All experiments were carried out at least thrice. 

***Statistical analysis***

The whole statistical analyses were performed by SPSS19 (Statistical Package for Social Science version 19, Inc., Chicago, Illinois, USA). For each method, we used three triplicated independent experiments. The results demonstrated as the mean±SD (standard deviation). Applying a one-way ANOVA t-test, we evaluated the statistical significance of discrepancy between the test and control. 

## Results

***Sequence analysis and physiochemical characterization of ent A-col E1 peptide***

**Figure 1:**
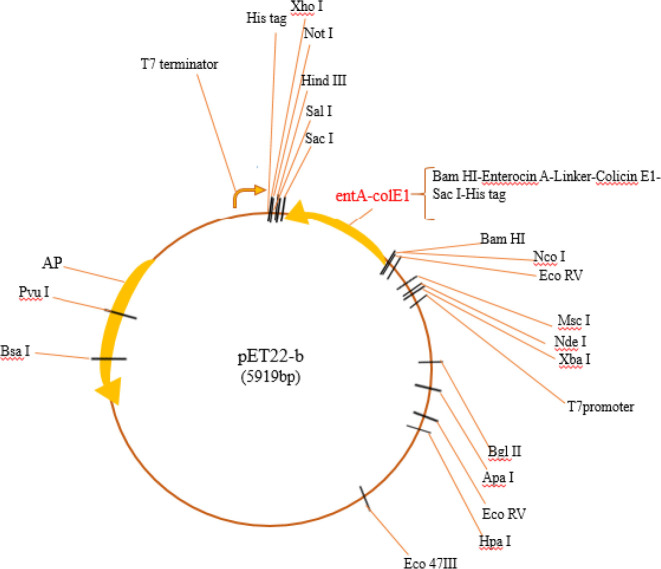
Pet22b vector including ent A-col EI peptide sequence

**Figure 2:**
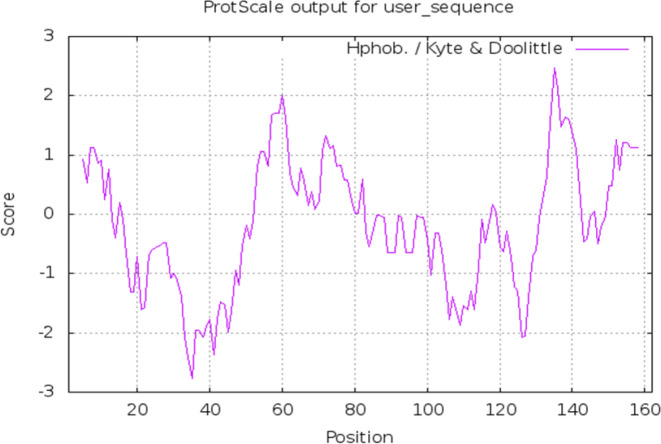
The Protscale output chart by using the Kyte & Doolittle algorithm. Peaks represent hydrophobic amino acids and valleys represent hydrophilic amino acids

pET22-b vector including the ent A-col EI peptide sequence is shown in Figure 1. Physiochemical characterization was determined by the Expasy’s ProtParam server shown in Table 1**.** Also, the hydrophobicity plot was drawn through the Kyte & Doolittle algorithm by protscale website (Figure 2).

***Secondary and tertiary structure prediction***

**Figure 3:**
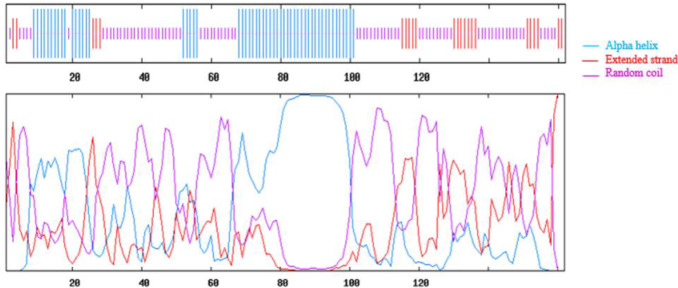
The predicted secondary structure of recombinant peptide by GOR4 server; the statistics are as follows: total residues: Alpha helix: 55, 33.95%, Extended strand: 23, 14.20%. Random coil: 84, 51.85%

**Figure 4:**
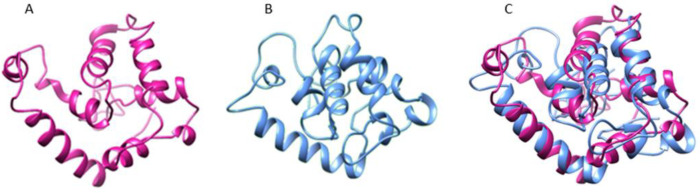
(A) The 3D structure of the truncated form, predicated by the I-TASSER server. (B) Schematic of protein after 100 nanoseconds simulation (this image was provided by Chimera software). (C) Modeled protein (pink) and simulated protein (blue) after 100 nsec. After being superimposed by Chimera software, it indicates that the structure is more impacted during the simulation period

**Figure 5.**
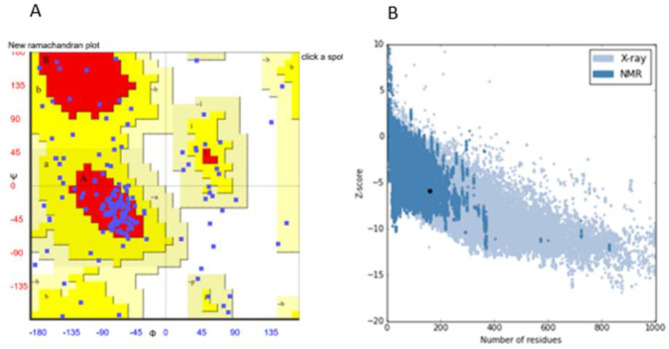
(A) Ramachandran plots for the predicted 3D structure of recombinant peptide created by RAMPAGE. Plot statistics indicate the number of residues in favored regions: 101 (63.1%), the number of residues in allowed regions: 36 (22.5%), and the number of residues in outlier regions: 23 (14.4%). (B) ProSA-web analysis: The Z-score of this protein is shown in a large black spot. The Z-score plot consists of the Z-scores of all experimental protein chains in PDB determined by NMR spectroscopy (dark blue) and X-ray crystallography (light blue)

Analysis of the secondary structure of ent A-col E1 peptide was performed by GOR4 server that predicted the arrangements of the amino acids as α-helices, β-sheets, and coils structures at specified positions. Result of this analysis is shown in Figure 3.  The tertiary structure of truncated ent A-col E1 peptide was modeled by the I-TASSER server. The server produced various 3D models, which between them the best pattern of this protein matching to the C-score (C-score=-2.89) was selected for further analysis. C-score value is based on the significance of threading template alignments, with values typically in the range of -5 to 2, where a higher C-score suggests a model with higher confidence, and -2.89 is significant. The 3D structure of truncated ent A-col E1 peptide is shown in Figure 4A. We used this model to simulate. Validation of this model with the rampage site showed that 85.6 % of the amino acids were within the permissible range of the Ramachandran diagram Figure 5A. The detected Z-score for all the modeled protein (-5.9) was within the typical range of the same size native proteins. This shows the quality of the model was well enough in Figure 5B.

***Molecular dynamic simulation***

**Figure 6:**
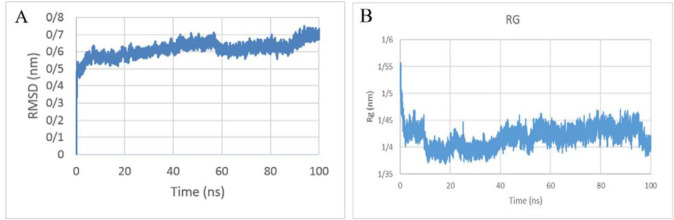
(A) RMSD of the chimeric protein during 100 nsec MD simulation. (B) Radius of gyration of the chimeric protein during 100 nsec MD simulation. RMSD: Root-mean-square deviation, MD: Molecular dynamics

Schematic of protein after 100 nsec simulation is shown in Figure 4B.Then, protein structure superimposed, which indicates that the structure is more impacted during the simulation period (Figure 4C). According to the graph, the RMSD protein oscillation value is about 0.6 nm. This protein is in equilibrium at about 40 nsec and remains at a fixed average RMSD until the end of the simulation. Therefore, it can be said that the protein has acceptable stability in Figure 6A. As shown in Figure 6B, this value is fixed for our protein after about 30 nsec and the structure becomes more compact during the simulation period.

***Expression and purification of ent A-col E1 peptide***

**Figure 7:**
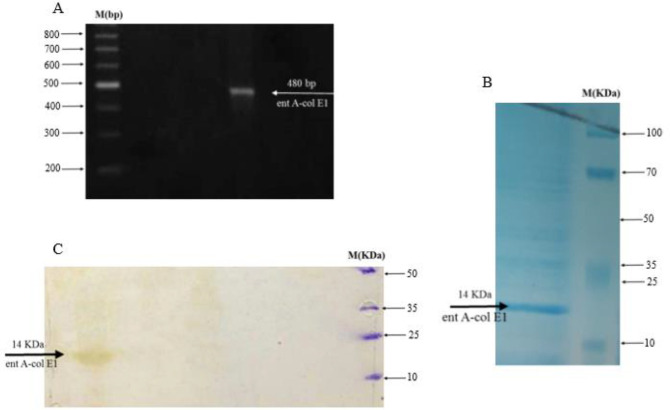
(A) Agarose gel electrophoresis for colony PCR product of transformed *Escherichia coli* BL21. (M) Marker. (B) Expression analysis of ent A-col E1 derived from the soluble phase of *E. coli* cell lysate, by SDS-PAGE (10%). M: Molecular weight markers. (C) Western blot analysis of the expressed ent A-col E1 peptide reacted with anti-His tag mAb. M: molecular weight markers. The arrow indicates the position of the overexpressed ent A-col E1 recombinant peptid

The ent A-col E1 peptide was transformed into the BL21 and was confirmed with observation of 486 bp band in gel electrophoresis by colony PCR with designed primers (Figure 7A). Then, the A-col E1 peptide was optimally expressed in *E. coli *BL21 and purified by a nickel column chromatography. The overexpressed fusion protein was obtained at 1 mM concentration of IPTG and 24 hr incubation. As shown in Figure 7B, the expressed ent A-col E1 peptide with a molecular weight of approximately 15 kDa was confirmed by SDS-PAGE and Western blot (Figure 7C). 

***Disk diffusion assay***

Our results demonstrated that the fusion peptide has antibacterial function against *S. aureus*, *E. coli, P. aeruginosa, *and* E. faecalis.* The inhibition zone of these bacteria growth was observed by disks saturated with different concentrations of ent A-col E1 peptide, (10, 30, and 110 μg/ml). This observation was in a match with the lytic action of standard antibiotics. However, an acceptable halo was not observed at the concentration of 5 μg/ml (Table 2). 

***Minimum inhibitory concentration assays***

MIC of the ent A-col E1 peptide is 10 μg/ml for *S. aureus* and *E. coli* and 20 μg/ml for *P. aeruginosa *and* E. faecalis*. While by reducing concentration of ent A-col E1 peptide to values ​​less than MIC, no antibacterial properties were observed.

***Time–kill curve analysis***

**Figure 8:**
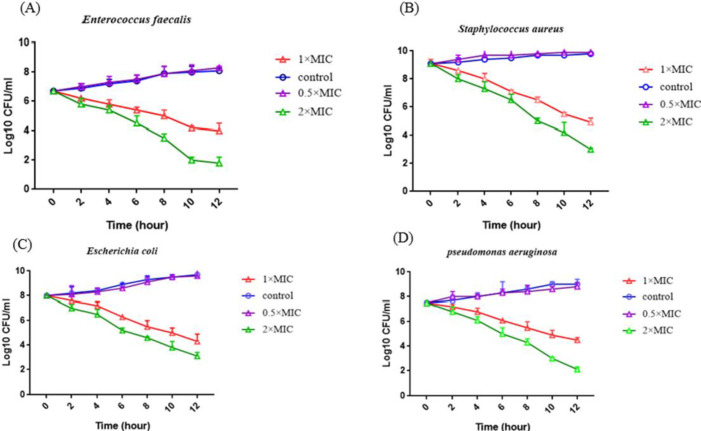
Time kill diagram to show the antimicrobial activity of the recombinant peptide against *Enterococcus faecalis* (A); *Staphylococcus aureus* (B); *Escherichia coli* (C); *Pseudomonas aeruginosa* (D); and untreated control

Based on the results of the kill assay, There was no significant reduction in the number of colonies of samples that were treated with 0.5 × MIC concentration of ent A-col E1. But, with the use of 1 and 2×MIC concentration of ent A-col E1, greater than 3 log_10 _-fold decrease in CFU was observed, which is equivalent to 99.9% killing of the inoculated bacteria. With increasing incubation time, the killing effect of the ent A-col E1 peptide also increased, and a decrease was observed in the number of colonies grown on the culture medium. By the end of the 12-hr incubation period, the number of bacteria grown on the culture medium decreased significantly. Profiles of killing were determined by a rapid and notable decline in bacterial count within the first 2 hr in the presence of fusion peptide (Figure 8).

***Effects of NaCl, heat, and pH treatments on the ent A-col E1 peptide activity***

**Figure 9:**
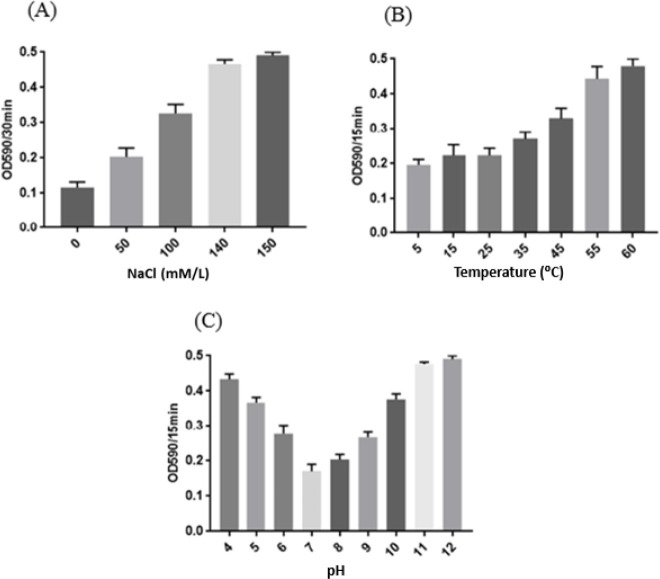
(A) Effect of different NaCl concentrations on ent A-col E1 peptide activity against *Staphylococcus aureus*. (B) Effect of various temperatures on ent A-col E1 peptide activity against *S. aureus*. (C) Effect of various pH on ent A-col E1 peptide activity against *S. aureus*

Ent A-col E1 peptide preserved its antibacterial function in up to 140 mM of NaCl. Our results showed that declining in the NaCl concentration from 140 to 0 mmol/l leads to enhancing the antibacterial activity of ent A-col E1 peptide. There was not any remarkable change in the activity of ent A-col E1 peptide under various temperatures, ranging from 5 to 45 ^°^C, for 15 min. Our findings illustrated that the optimal activity of ent A-col E1 peptide was at 5–35 ^°^C and pH 6-8, but the protein also exhibited favorable activity at pH values from 6 to 9 (Figure 9). 

**Table 1:** The computed physicochemical characterization using the expasy’s protparam tool

**Value**	**Physiochemical parameters**
144	Number of amino acids
14054.33	Molecular weight
8.90	Theoretical isoelectric point (IP)
30 hr (mammalian reticulocytes, *in vitro*) >20 hr (yeast, *in vivo*) >10 hr (*Escherichia coli*, *in vivo*)	Estimated half-life
25.11	Instability index (II)
77.35	Aliphatic index
-0.120	Grand average of hydropathy (GRAVY)

**Table 2:** Antimicrobial activity of the recombinant peptide by disk diffusion assay

Pathogen Name	Positive control (Zone of inhibition)	Recombinant peptide (Zone of inhibition)	Negative control (Zone of inhibition)
*Staphylococcus aureus*	(Penicillin) 33±1	25±0.7	0.0
*Escherichia coli*	(Cefazolin) 18±1.5	19±1	0.0
*Pseudomonas aeruginosa*	(Piperacillin/tazobactam ) 28±0.8	22±0.5	0.0
*Enterococcus faecalis*	(Ampicillin) 21±2	18±1.2	0.0

Values are mean±SD of three independent experiments performed in triplicates, the zone of inhibition was measured in millimeter (mm)

## Discussion

Bacteriocins are peptides with bactericidal or bacteriostatic activities, which are ribosomally synthesized in some Gram-positive and -negative bacteria (29). Enterocins produced by *Enterococci* and colicins produced by some *E.* *coli* strains have antimicrobial activities against food-spoilage, food-born, and human pathogenic bacteria (14, 30). Enterocin A-Colicin E1 recombinant protein is capable of production and purification at high levels. Research on enterocin A and colicin E1 has been performed separately, but their fusion protein composition has been investigated for the first time in this study and antibacterial activity was investigated by modifying two bacteriocins.

In the current study, we proposed bioinformatics analysis, expression, purification, and antibacterial activity of a new fusion peptide containing enterocin A and colicin E1. This designed small peptide had proper stability in different conditions and acceptable function against experimented bacterium strains. The cysteine amino acid linker sequence, inserted between the two bacteriocin domains of the construct, was used to increase the solubility of the protein as well as to increase the antibacterial activity and cleavage by the proteases to prevent side complications. Due to the preservation of the recombinant protein structure, codon usage for expression in *E. coli* was not performed. This 144-amino-acid sequence was produced as a stable and functional protein. To investigate the structure in detail, the I-TASSER server was employed in the current study. At present, the best and powerful predictive server of 3D protein structure is the I-TASSER server, which automatically models proteins with less than 1500 amino acids by threading method. It also provides valuable information about the secondary structure of the protein, access to the solvent, and possible active site. This server uses 9 threading programs to trace the 3D structure to find the best template. After reviewing several indicators for alignment, the consensus areas were designed with the modeler program. Analysis of the 3D structure of the peptide indicated that the model 1 structure with a c-score of -2.89 has greater functional properties compared to other models that is probably the most suitable form to bind to bacterial cell walls. Ramachandran plot analysis supported the suitability and sustainability of modeled peptides indicating that about 85.6% of residues were desired and had sterically-allowed regions. All the data are compelling evidence for the functionality of the fusion peptide. Also, the modeled peptide had an acceptable Z-score similar to that of natural proteins indicating that the design had good quality. Simulation of the data showed that after 100 nsec, the protein has a greater impression and stability. The RMSD diagram also showed that the designed peptide had acceptable stability. The gyration radius as an important factor indicates the stability of the protein in the simulation environment. Thus, if the protein is well-stable in the simulation medium, the average gyration radius for that protein during the simulation period is a constant value. This value is fixed for our protein after about 30 nsec, and the structure becomes more compact during the simulation period. Hence, the ent A-col E1 peptide had a proper functional structure based on the bioinformatics predictions. Bacteriocin, a good food-preservative, and the antimicrobial agent have recently drawn researchers’ attention (5). However, due to their low production by native strains, bacteriocins are not commercially applicable. Also, some Enterococci are considered opportunistic pathogens that cause various infections. Hence, researchers focus on other intestinal microflora such as *E. coli* in terms of the production and release of bacteriocins (31). Different hosts express an active form of bacteriocins, e.g., enterocin P and enterocin L50; however, the level of expression is insignificant (32, 33). PET 22b vector containing the N-terminal pel B secretion signal was employed in the current study. This signal sequence can ideally be used as a strategy to direct the expression of bacteriocins to the periplasm in *E. coli* (34). *E. coli* periplasm disulfide oxidoreductases and isomerases catalyze soluble peptides in the desired amount; hence, such therapeutic peptides can be presented from the periplasm as an ideal place for this target (35). Protein secretion is directed by the PelB leader sequence in the pET-22b vector to the bacterial periplasm that can prevent inclusion bodies in the cytosol; it leads to soluble protein expression in higher extents. SDS-PAGE results in the current study showed that the expression of protein from *E. coli* lysate in the soluble phase was significant, which increases protein activity and improves their function. Among the reported enterocins, enterocin A rarely shows the broad-spectrum antimicrobial activity. However, the observed activity against Gram-negative organisms is unusual and has thus far been reported for only a few bacteriocins produced by LAB (36, 37). In the present study, both Gram-negative and Gram-positive strains were used and acceptable antibacterial activity of ent A-col E1 against different bacteria strains was observed. In the disk diffusion method, the inhibition zone diameter caused by entA-colE1 peptide was compared to the inhibition zone caused by common antibiotics of each strain, most of which were either the same size or close to the size of the halo created by the common antibiotics. Degradation of nucleic acid, permeabilization of the cytoplasmic membrane, or inhibition of protein synthesis are the mechanisms through which bacteriocins exert their inhibitory effects on bacteria (38). Pore-forming colicins in Gram-negative bacteria bind to the outer membrane receptors, translocate across the periplasmic space, and enter into the cytoplasmic membrane to form a highly-conductive ion channel (39, 40). A- and B-type enterocins are molecules that disrupt peptidoglycan layer synthesis; they can kill target cells through binding to the lipid II molecule and inhibition of cell wall synthesis (41). Enterocin A can also kill the target bacteria by binding to the lipid II molecule and forming pores in the cytoplasmic membrane. The pore formation of enterocin A is the major killing mechanism (42). In a study by Ankaiah *et al.*, the antibacterial and anti-biofilm activities of the recombinant peptide composed of enterocins A and B were compared to those of enterocin B and the results showed that the fusion peptide had higher antibacterial activity compared to enterocin B (20). Moreover, another study demonstrated that the bioengineered nisin A derivatives had increased activity against both Gram-positive and Gram-negative pathogens (19). The low values of MIC obtained in the present study indicate the good antibacterial activity of ent A-col E1 against both Gram-positive and Gram-negative strains, which increased with increasing time up to 24 hr. Similarly, Beric *et al.* showed that the antibacterial function of licheniocin increases with increasing the exposure time (43). Literature data about MIC for recombinant bacteriocins are scarce. Antimicrobial peptides might be inactivated by physiological salt, which is the major weakness of such molecules for clinical applications (44). The antibacterial activity of ent A-col E1 maintained in 140 mM NaCl, while gramicidins, human defensin-1, magainins, and bactenecins lost their properties under the same conditions as reported by different studies (45). Myxinidin can show antimicrobial activities even in high-salt solutions against various bacteria, consistent with the current study findings (46). Furthermore, the investigation of activity maintenance strongly suggested that ent A-col E1 peptide is stable in a wide spectrum of temperatures from 5 to 45 ^°^C and can maintain proper folding. This recombinant peptide can well-tolerate pH alterations and obtain a specific active state, which is its advantage over other common bacteriocins; nisin is highly heat-resistant but sensitive to alkaline conditions (47). Therefore, ent A-col E1 peptide has promising implications to treat bacterial infections due to its good stability and antibacterial activities. 

## Conclusion

The results of bioinformatics design and antimicrobial analyses presented the novel arrangement of functional enterocin A and colicin E1. The purified fusion peptide demonstrated favorable characteristics and specific function under several thermal, pH, and saline conditions. We observed considerable antibacterial activity of ent A-col E1 against *S. aureus*, *E. coli*, *P. aeruginosa, *and* E. faecalis*. All of these findings favor the notion that ent A-col E1 may suggest behest for the development of an agent with effective antibacterial properties in the future. However, further investigation is needed to prepare more evidence on various aspects of the antibacterial function of this fusion peptide. In this regard, it is recommended that further studies be performed to compare the effect of ent A-col E1 with each of colisin E1 and enterocin A separately. 

## Funding

This research was supported by the Kashan University of Medical Sciences (Grant no. 97149). The funding agency had no role in the design of the study and collection, analysis, and interpretation of data and in writing the manuscript. 
